# Hypothermia Activates Adipose Tissue to Promote Malignant Lung Cancer Progression

**DOI:** 10.1371/journal.pone.0072044

**Published:** 2013-08-27

**Authors:** Gangjun Du, Bei Zhao, Yaping Zhang, Ting Sun, Weijie Liu, Jiahuan Li, Yinghui Liu, Yingying Wang, Hong Li, Xidong Hou

**Affiliations:** Institute of Pharmacy, Pharmaceutical College of Henan University, Jinming District, Kaifeng, Henan, China; Cincinnati Children's Hospital Medical Center, United States of America

## Abstract

Microenvironment has been increasingly recognized as a critical regulator of cancer progression. In this study, we identified early changes in the microenvironment that contribute to malignant progression. Exposure of human bronchial epithelial cells (BEAS-2B) to methylnitrosourea (MNU) caused a reduction in cell toxicity and an increase in clonogenic capacity when the temperature was lowered from 37°C to 28°C. Hypothermia-incubated adipocyte media promoted proliferation in A549 cells. Although a hypothermic environment could increase urethane-induced tumor counts and Lewis lung cancer (LLC) metastasis in lungs of three breeds of mice, an increase in tumor size could be discerned only in obese mice housed in hypothermia. Similarly, coinjections using differentiated adipocytes and A549 cells promoted tumor development in athymic nude mice when adipocytes were cultured at 28°C. Conversely, fat removal suppressed tumor growth in obese C57BL/6 mice inoculated with LLC cells. Further studies show hypothermia promotes a MNU-induced epithelial-mesenchymal transition (EMT) and protects the tumor cell against immune control by TGF-β1 upregulation. We also found that activated adipocytes trigger tumor cell proliferation by increasing either TNF-α or VEGF levels. These results suggest that hypothermia activates adipocytes to stimulate tumor boost and play critical determinant roles in malignant progression.

## Introduction

Since the signing of the National Cancer Act in 1971, cancer has remained a major cause of death despite significant progress in understanding its biology and treatment [1]. During the past decades, advances in identifying aberrances in oncogenes and tumor suppressor genes within tumor epithelial cells caused the role of the microenvironment in tumorigenesis to be overlooked [2]. The phenotypic and genotypic abnormalities in cancer epithelial cells cannot fully delineate tumor phenotypes and clinical behavior [3], and in fact, there is increasing evidence that the microenvironment is an active participant throughout cancer initiation, progression, and metastasis [4]. Numerous studies have demonstrated that tumor cells already carrying critical genetic alterations can remain dormant or be triggered to proliferate by changes occurring in their microenvironment [5]. There is also strong evidence that microscopic tumors are commonly present in adults in the form of dormant lesions [6]. A subsequent switch from dormancy to aggressive proliferation may take several years to decades. Thus, tumor lesions may be maintained in an initially non-permissive microenvironment but transition to a proliferative state due to extrinsic changes within the microenvironment [7]. A better understanding of the mechanisms that regulate the switch would not only allow for more accurate identification of patients that can benefit from systemic therapy but can also lead to the development of more targeted therapies for inhibiting the signals that promote disease progression.

Recent studies have postulated that tumors can be kept in check for long periods through a dynamic balance that results in the progressive loss of immunogenicity by tumor cells [8]. Tumor initiation first needs to escape extinction in a stochastic birth–death proliferation process. Next, the transformed cells exist in a quiescent state for many years or, alternatively, as dormant tumor cells whose cellular proliferation is balanced by apoptosis. Finally, the dormant tumor can progress to clinical disease once a growth factor-favorable microenvironment is activated to support continued tumor growth [9]. The critical triggers that regulate this transition from dormant tumor cells into proliferative ones that lead to disease progression remain unknown.

We hypothesized that hypothermia favors the epithelial-mesenchymal cell transition and stresses apoptotic escape. Hypothermia is often associated with compromised host defenses and provides an adaptive mechanism for stress tolerance, allowing cells to survive non-physiologic conditions [10]. However, it is also possible that the same adaptive mechanism can ultimately favor malignant transformation by interfering with pathways that regulate cell growth and apoptosis. The dual character of this response is supported by the increase in the formation of micronucleated polychromatic erythrocytes in mouse bone marrow under long-lasting hypothermia [11], while low temperatures have also been shown to protect mammalian cells from apoptosis initiated by various stimuli [12].

We also considered other factors that may play a major role in the transition from tumor cell dormancy to proliferation. Because obesity is associated with an increased risk and poor prognosis for many types of cancer and because cold exposure is considered a critical factor for adipose tissue activation [13]–[15], we proposed that the combination of a hypothermic environment and adipose tissue activation promotes malignant progression. To test this hypothesis, we observed the relative contributions of hypothermia and adipose tissue activation on carcinogenesis with the goal of (a) identifying the early changes in microenvironment that contribute to malignant progression and (b) developing new therapeutic strategies that tackle the microenvironment to eradicate tumors or, at least, maintain tumor dormancy and transform cancer into a chronic disease.

## Results

### Hypothermia suppressed MNU-mediated cytotoxicity and promoted cell clonogenic capacity in vitro

**Figure 1:**
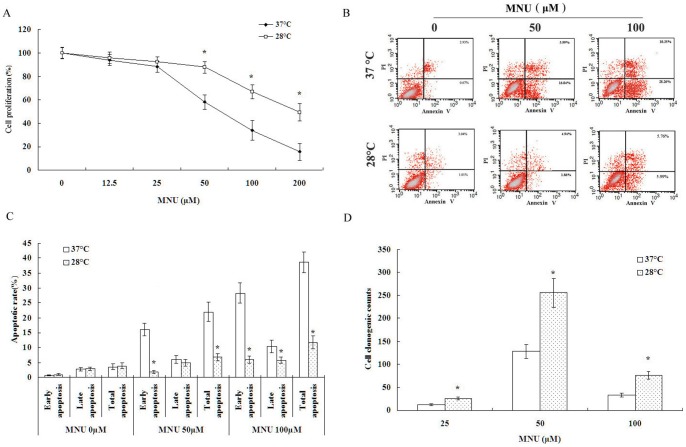
Hypothermia suppressed MNU-mediated cytotoxicity and promoted cell clonogenic capacity. A. Hypothermia suppressed MNU-induced BEAS-2B cytotoxicity examined by MTT. B and C. Hypothermia decreased MNU-induced BEAS-2B cell apoptosis examined by PI and Annexin V-FITC staining, early apoptosis and late apoptosis were determined as the percentage of Annexin V+/PI- cells and Annexin V+/PI+ cells, respectively. D. Hypothermia promoted MNU-induced BEAS-2B cell clonogenic capacity examined by soft agar assay. Data were expressed as mean ± SD. One asterisk (*)<0.001 as compared to 37°C condition (n = 5).

The BEAS-2B cell line is a reasonable model cell for toxicological studies because it expresses differentiation characteristics of human lung explants [20]. MNU is a direct-acting alkylating agent that interacts with DNA. At physiological pH, MNU spontaneously decomposes to form a carbonium ion which is capable of alkylating the nitrogens and oxygens of DNA bases by a Snl reaction [21]. This chemical was initially dissolved at 100 times the desired concentration in citrate buffer (pH 4.5) and then diluted appropriately in serum-free medium. With regard to thermolabile property of MNU, alkylation continued throughout the entire 2-h exposure interval, which is proved that under the conditions the number of gaps detected in nascent DNA of alkylated cells were not influenced [22]. Mammalian cells cultured in vitro are able to recover from cold stress. The cellular structures examined were, in general, well maintained during mild hypothermia (27–32°C) but became increasingly disrupted at low temperatures (4–10°C) [23]. Based on maximal tolerance to low temperature which still allowed the majority of cells to survive and proliferate although cell growth was decreased, we examined the effects of a hypothermic environment (28°C) on human bronchial epithelial cells (BEAS-2B) exposed in vitro to the direct carcinogenic agent methylnitrosourea (MNU). Lowering the culture temperature from 37°C to 28°C significantly suppressed MNU-mediated cytotoxicity in cultured BEAS-2B cells over a 48-h period (Fig. 1A). This observation was supported by PI and Annexin V-FITC staining (Fig. 1B and C). Unexpectedly, in MNU-treated BEAS-2B cells, the decrease in temperature from 37°C to 28°C increased clonogenic capacity (Fig. 1D).

**Figure 2:**
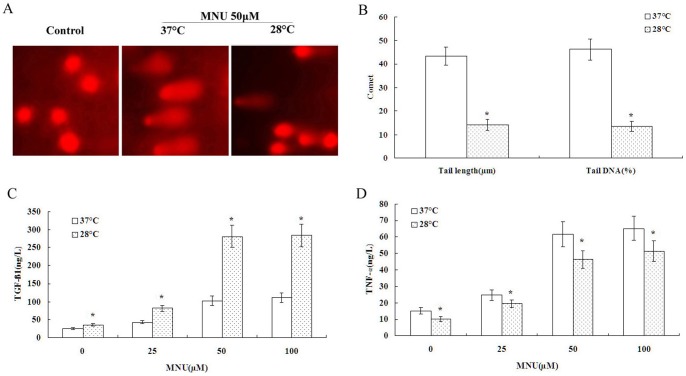
Hypothermia suppressed MNU-mediated DNA demage by TGF-β1 unregulation and TNF-α down-regulation. A and B. Hypothermia decreased MNU-induced BEAS-2B cell DNA demage examined by comet assay. C. Hypothermia increased TGF-β1 level examined by ELISA in MNU-treated BEAS-2B cells. D. Hypothermia decreased TNF-α level examined by ELISA in MNU-treated BEAS-2B cells. Data were expressed as mean ± SD. One asterisk (*)<0.001 as compared to 37°C condition (n = 5).

**Figure 3:**
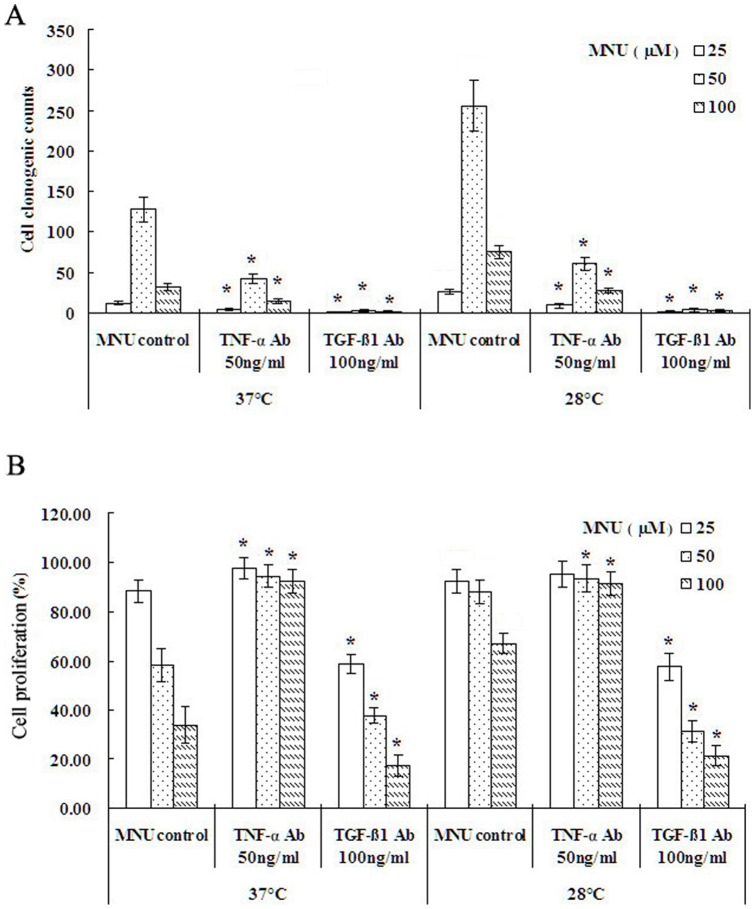
Effect of neutralization of TNF-α or TGF-β1 on colony formation and cytotoxicity. A. Neutralization of TNF-α or TGF-β1 by a blocking antibody reduced MNU-induced clonogenic capacity under 37°C and 28°C condition. B. Neutralization of TNF-α abrogated MNU-induced cytotoxicity but neutralization of TGF-β1 promoted MNU-induced cytotoxicity under 37°C and 28°C condition. Data were expressed as mean ± SD. One asterisk (*)<0.001 as compared to MNU control under the same condition (n = 5).

The DNA damage induced by MNU is considered to be characteristic of lesions produced in DNA by alkylating agents. Inflammatory responses play a vital role at different stages of chemical carcinogenesis, including initiation, progression and recurrence. TNF-α has been recently shown to promote tumor development in experimental carcinogenesis, and TGF-β has been shown to attenuate an anti-tumor immune-response through the induction of regulatory T cells in spontaneous and inflammation associated cancer [24]. However, excessive inflammation can play a beneficial role in response to tumor damage although the effects of chronic inflammation are sufficient to initiate cancer transformation and development [25]. Obviously, the critical balance between TGF-β and TNF-α might have a key role on tumor transformation. To better understand the relationship between a hypothermic environment and cytotoxicity, we carried out a comet assay and TNF-α and TGF-β1 ELISAs. A significant reduction of total DNA damage was observed in BEAS-2B cells exposed to 50 μM of MNU at 28°C compared to 37°C (Fig. 2A and B). Interestingly, the decrease in temperature from 37°C to 28°C reduced TNF-α levels and increased TGF-β1 levels in MNU-treated BEAS-2B cells (Figure 2C and D). Neutralization of TNF-α by a blocking antibody reduced MNU-induced clonogenic capacity and abrogated MNU-induced cytotoxicity (Fig. 3A and B). Conversely, the neutralization of TGF-β1 resulted in few cell colonies and promoted MNU-induced cytotoxicity (Fig. 3A and B). These results suggest that a balance between TGF-β1 and TNF-α determine cell rest (slope to TGF-β1), death (slope to TNF-α), or proliferation (balance).

### Hypothermia-incubated adipocyte media promotes clonogenic capacity in MNU-treated BEAS-2B cells and proliferation in A549 and LLC cells

**Figure 4:**
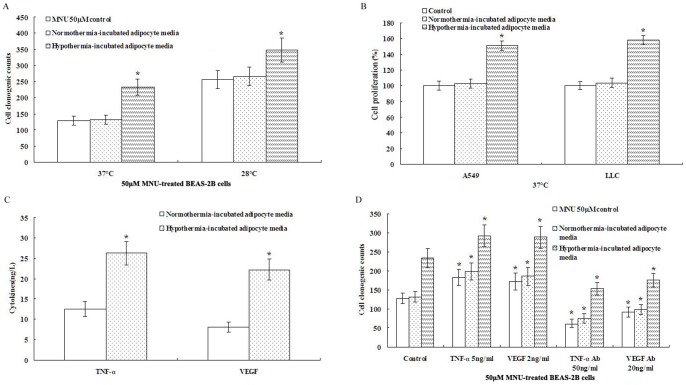
Hypothermia-incubated adipocyte media promotes clonogenic capacity in MNU-treated BEAS-2B cells and proliferation in A549 and LLC cells. A. Hypothermia-incubated adipocyte media promoted clonogenic capacities by soft agar assay under the same temperature condition in MNU-treated BEAS-2B cells. B. Hypothermia-incubated adipocyte media promoted proliferation examined by MTT in A549 and LLC cells at 37°C. C. The levels of TNF-α and VEGF were elevated in hypothermia-incubated adipocyte media. D. Addition of TNF-α or VEGF to adipocyte media promoted clonogenic capacities and TNF-α and VEGF blocking antibodies prevented the clonogenic capacities relative to control under the same media condition. Data were expressed as mean ± SD. One asterisk (*)<0.001 as compared to normothermia-incubated adipocyte media (n = 5).

Tumor-associated stroma is typified by a persistent, non-resolving inflammatory response that enhances tumor angiogenesis, growth and metastasis. The inflamed tumor-associated adipose tissue fuels the growth of malignant cells by acting as a proximate source for vascular endothelium and activated pro-inflammatory cells [26]. The adipose tissue hyperplasia is a fundamental response to low ambient temperature, at the same time, the major inflammatory adipokines, TNF-α and VEGF, increased in adipose tissue [27], [28]. Based on these observations, we tested the hypothesis that the combination of a hypothermic environment and adipocyte activation determines malignant progression. We first observed the contribution of adipocytes to cancer progression. Supernatants (media) from differentiated adipocytes cultured at 37°C and 28°C were used to treat cells (final concentration was diluted ten fold). As shown in Fig 4, hypothermia-incubated adipocyte media promoted clonogenic capacities in MNU-treated BEAS-2B cells and proliferation in A549 and LLC cells (Fig. 4A and B). In this study, A549 and LLC cells were selected because A549 cell line is derived from alveolar epithelial type II cells and LLC cell line is originated spontaneously as a carcinoma of the lung of a C57BL mouse, which express most of the major components in lung epithelial cells [20], [29]. The purpose of this study was to evaluate whether hypothermia-activated adipocytes promoted tumor transformation of normal cells (BEAS-2B) and accelerated the presence of tumor.

Next, we considered the possible involvement of adipokines, which are known to physiologically regulate energy balance. We found that the levels of TNF-α and VEGF were significantly elevated in hypothermia-incubated adipocyte media compared to normothermia-incubated adipocyte media (Fig. 4C). To ascertain whether TNF-α and VEGF play functional roles in hypothermia-activated adipocytes, we tested the effect of adding or neutralizing these factors on clonogenic capacities in MNU-treated BEAS-2B cells. TNF-α and VEGF blocking antibodies prevented the clonogenic capacities (Fig. 4D). Conversely, the addition of TNF-α or VEGF to adipocyte media promoted clonogenic capacities (Fig. 4D). In addition, the effects of TNF and VEGF blockade appear to be proportional in all groups, including the controls, this implys that the TNF-α and VEGF blockade are acting independently of the conditioned media. However, the clonogenic promotion of hypothermia-incubated adipocyte media was also significantly correlated with expression of TNF-α and VEGF, therefore, we conclude that hypothermia may activate adipocytes to promote cancer progression partially by increasing TNF-α and VEGF levels.

### A hypothermic environment activated adipocytes and promoted tumor proliferation

**Figure 5:**
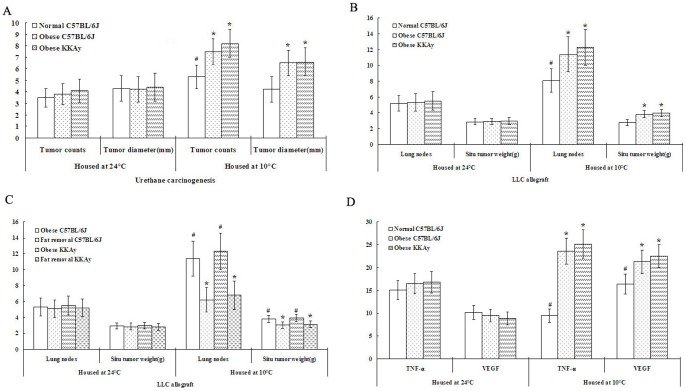
Hypothermia activated adipocytes and promoted tumor proliferation. A and B. The hypothermia alone increased numbers of primary tumors and metastases but had no effect on tumor diameter in normal mice, whereas the obese mice relative to normal C57BL/6J mice showed more tumor counts and bigger tumor size at 10°C but not at 24°C in urethane carcinogenesis and LLC allograft, respectively. C. The fat removal mice relative to obese mice showed less metastatic lung nodes and smaller situ tumor at 10°C but not at 24°C in LLC allograft. D. The obese mice relative to normal C57BL/6J mice showed more serum TNF-α and VEGF at 10°C but not at 24°C. Data were expressed as mean ± SD. One asterisk (*)<0.001 as compared to normal mice housed at 10°C and one well (#)<0.001 as compared to normal mice housed at 24°C (n = 10).

Accumulation of mutations may enhance cancer risk in target organs or cause cell death in susceptible tissues or cells when excessive DNA damage is not repaired. Although MNU-induced carcinogenesis can be used as organ-specific animal models for human cancer [30], we could not use this carcinogenic agent to induce lung cancer in murine. In contrast to MNU, despite low cytotoxicity in vitro, urethane has been most extensively utilized for the induction of lung cancer in mice [31]. In addition, the LLC cell line is also a well-established mouse cancer model that is commonly used as a transplantable malignancy model in syngeneic C57BL/6 mice and seems highly relevant to clinical settings [32]. To support these in vitro data, mice were housed in a normothermic environment (24°C) or in a hypothermic environment (10°C) after they were either injected intraperitoneally with urethane at 800 mg/kg once weekly for 8 weeks or inoculated subcutaneously with Lewis lung cancer (LLC) cells. As shown in Fig. 5A and B, the hypothermia increased numbers of primary tumors and metastases in normal and obese mice. Although environmental hypothermia promoted urethane-induced lung tumor counts in all breeds of mice (all 20 mice in each group developed lung tumors), an increase in tumor size could be discerned only in obese mice housed in a hypothermic environment (Fig. 5A). Similarly, even though environmental hypothermia promoted metastatic lung nodes in all breeds of mice inoculated subcutaneously with LLC cells (all 10 mice in each group developed metastatic lung nodes), accelerated growth of the in situ tumor could again only be observed in obese mice housed in a hypothermic environment (Fig. 5B). On a separate note, genetic- and diet-induced obesity neither influenced urethane-induced tumor yield nor affected inoculated tumor progression in mice housed in a normothermic environment (Fig. 5A and B). To further verify this observation, obese mice with inguinal fat removal were inoculated with LLC cells. Fat removal significantly reduced hypothermia-induced tumor growth and metastasis (Fig. 5C). Serum ELISA for TNF-α or VEGF revealed that a hypothermic environment significantly increased TNF-α and VEGF levels in obese mice (Fig. 5D).

Exposure of humans and rodents to cold activates thermogenic activity in adipose tissue that could counteract hypothermia. The ability to produce heat through UCP1-mediated adaptive nonshivering thermogenesis is considered to be a critical evolutionary development that promoted the radiation of eutherian mammals in cold environments [33]. In this study, hypothermic environment (10°C) was selected because this was the threshold ambient temperature at which none of the animals suffered from frostbite or any other adverse side effects resulting from cold exposure, if less than 7°C, it led to premature deaths in mice. During the experiment, the ambient temperature was restricted to 8–12°C to ensure the normal internal body temperature (>36°) by a specially converted refrigerator cabinet fitted with a perspex door and appropriate shelving. The mice under hypothermic condition ate consistently more food throughout the experiment and kept normal physical activity.

These results suggest that although the tropic potential of hypothermia is limited, it is sufficient to promote carcinogenic incident and that adipocytes alone neither influences tumor yield nor affects tumor progression until it is activated by hypothermia.

### Hypothermia promotes the epithelial-mesenchymal transition induced by MNU and protects the tumor cell against immune control in vitro

**Figure 6:**
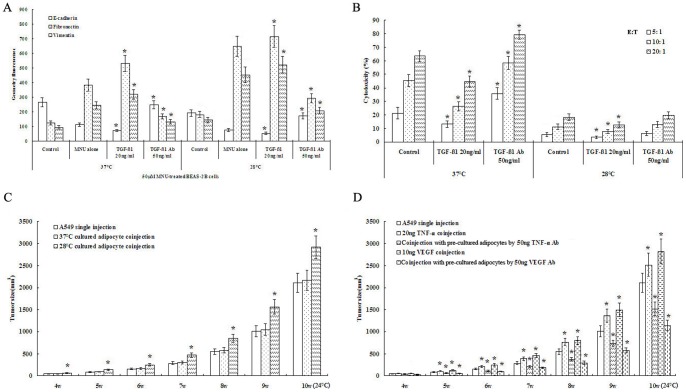
The hypothermia-activated adipocytes promoted lung cancer progression by TGF-β1 and TNF-α. A. TGF-β1 neutralization relative to MNU alone down-regulated epithelial marker E-cadherin and upregulated mesenchymal markers, such vimentin and fibronectin, and adding TGF-β1 to cells had opposite action in MNU-treated BEAS-2B cells (n = 5). B. TGF-β1 neutralization relative to control promoted CD8+ T cell-mediated cytotoxicity at 37°C and adding TGF-β1 to cells suppressed CD8+ cell-mediated cytotoxicity in A549 cells (n = 5). C. Xenografts of coinjection using A549 cells and 28°C-cultured adipocytes grew rapidly relative to A549 single injection (n = 10). D. A coinjection using either TNF-α or VEGF and A549 cells promoted xenograft development, whereas pre-cultured adipocytes by a TNF-α or VEGF blocking antibody prevented xenograft development relative to A549 single injection (n = 10). Data were expressed as mean ± SD. One asterisk (*)<0.001.

Epithelial-mesenchymal transition (EMT) is an important mechanism in carcinogenesis [34]. For early tumor development, the escape of the first lines of defense of the immune surveillance is also a critical step which determines survival or destruction [35]. To further prove the relationship between a hypothermic environment and chemicalcarcinogenesis, we observed effects of hypothermia on MNU- induced EMT in BEAS-2B cells and CD8+ cell-mediated cytotoxicity in A549 cells. Cultures at 28°C, relative to those at 37°C, down-regulated epithelial marker E-cadherin and upregulated mesenchymal markers, such vimentin and fibronectin, in MNU-treated BEAS-2B cells (Fig. 6A). Similarly, cultures at 28°C significantly reduced mouse CD8+ cell-mediated cytotoxicity in A549 cells (Fig. 6B). Since TGF-β1 is a multipotent cytokine that exert both EMT and immune evasion at different stages of carcinogenesis [36], we want to know whether TGF-β1 also plays functional roles in MNU-induced EMT and in immune cell-mediated cytotoxicity, we measured EMT and immune cell-mediated cytotoxicity in cells treated with a TGF-β1 blocking antibody. TGF-β1 neutralization promoted CD8+ T cell-mediated cytotoxicity at 37°C but had no effect it at 28°C (Fig. 6A and B). Conversely, adding TGF-β1 to cells promoted EMT in BEAS-2B cells and suppressed CD8+ T cell- mediated cytotoxicity in A549 cells (Fig. 6A and B). These results suggest that hypothermia-induced EMT, but not tumor immune evasion, is dependent on TGF-β1.

### Activated adipocytes promote tumor growth

Currently human tumor xenografts are the most widely used model to help predict antitumor efficacy in a preclinical setting and can invariably provide greater insight into the complex mechanisms that underlie the development and pathogenesis [37]. Using A549 xenograft model, we investigated the effect of adipocytes on tumor progression. When differentiated 3T3-L1 adipocytes were cultured at 37°C, a coinjection using adipocytes and A549 cells did not reveal an obvious host-tumor interaction in primary tumors in athymic nude mice housed at room temperature (24°C) (adipocytes alone resulted in no tumor incidence). However, xenografts of coinjection using adipocytes and A549 cells grew rapidly when differentiated 3T3-L1 adipocytes were cultured at 28°C for 24 h (Fig. 6C). To further evaluate the roles of TNF-α and VEGF for adipocyte activation, we also tested the effect of adding these factors to tumor cells or neutralizing these factors in adipocytes on A549 xenografts. A coinjection using either TNF-α or VEGF and A549 cells promoted xenograft development. Conversely, pre-cultured adipocytes by a TNF-α or VEGF blocking antibody prevented xenograft development (Fig. 6D). These results suggest that activated adipocytes promote tumor growth.

## Discussion

Clinical and experimental evidence suggest that human tumors can persist for long periods of time as microscopic lesions in a dormant state [12]. Epidemiology studies have shown that obesity increases the risk for many types of cancer, including lung cancer [13], [14], [38]. However, it remains unknown what role adipocytes play in the transition from tumor cell dormancy to proliferation and how adipocytes stimulate malignant progression. Here, we investigated the multiple ways adipocytes can uniquely influence the characteristics and phenotypic behavior of malignant lesions and lung cancer cells. Our study shows that hypothermia promotes EMT and tumor immune evasion by TGF-β1 dependent and independent ways, respectively, and that adipocytes activated by a hypothermic environment drive malignant progression by increasing TNF-α and VEGF levels.

The recent emergence of the tumor microenvironment as a critical determinant in cancer biology is paralleled by the promising therapeutic potential it carries by opening alternate routes for combatting cancer [3]. Tumor progression is a late step in carcinogenesis. Understanding the regulatory machinery of this progression is essential for identifying early changes in microenvironment that contribute to malignant progression and could provide a rationale for the development of novel agents for targeting dormant tumor cells.

Malignant lesions need immune escape and an energy source to expand. Conventionally, the energy source driving cancer cell proliferation results from aerobic glycolysis [39]. It is now becoming increasingly clear that cancer arises as a consequence of dysregulated metabolism in response to an altered energy status and endocrine factors [40]. Since energy is stored predominately as lipids in adipose tissue in distinct anatomical locations and cold exposure is a major regulatory factor for adipose activation [15], [41], we proposed that the combination of a hypothermic environment and adipocyte activation would produce suitable stromal niches for cancer progression. Consistent with our hypothesis, a hypothermic environment promoted TGF-β1 but reduced TNF-α; this translates into the transition from acute inflammation to chronic inflammation and also contributes to malignant lesions. Activated adipocytes, however, produced TNF-α and VEGF, both of which stimulate cell proliferation and supply an energy source for the tumor. Consequently, the combination of a hypothermic environment and adipocyte activation promoted malignant progression. These results led to the proposal of a novel carcinogenic model, termed the “cold carcinogenic effect.” In this model, the hypothermic environment generated by injury-induced ischemia and hypoxia produces a suitable niche for tumor lesions and then activates adjacent adipocytes to provide the proliferative factors (such as TNF-α and VEGF) and energy-rich nutrients (adipose metabolic products) for malignant progression.

Although we cannot measure temperature and adipose activation of regional tissues damaged by a carcinogenic agent in vivo, earlier studies revealed that hypothermia stimulates cell proliferation in the small bowel as well as in the proximal colon, where it has a correspondingly mild co-carcinogenic effect [42]. Conversely, local hyperthermia for various types of malignant tumors has shown promising antitumor effects [43]. Recent studies have also shown that obesity increases the risk of endometrial cancer and is linked to higher mortality rates in the general population [44], whereas surgical removal of the parametrial fat pads stimulates apoptosis and inhibits UVB-induced carcinogenesis [45]. Consistent with these findings, a hypothermic environment in this study increases clonogenic capacity in MNU-treated BEAS-2B cells, and activated adipocytes promoted clonogenic capacities in MNU-treated BEAS-2B cells and proliferation in A549 and LLC cells. However, our results indicate that genetic- and diet-induced obesity alone neither influenced urethane-induced tumor yield nor affected inoculated tumor progression in mice housed in a normothermic environment, suggesting that the obesity-dependent carcinogenic effect relies on hypothermic activation.

The heat shock proteins (Hsps) play crucial roles in environmental stress tolerance and in thermal adaptation. Elevated levels of certain HSPs in tumor cells could have an inhibitory effect on apoptosis and therefore protect tumour cells from death. At the same time, increased levels of HSPs in tumor cells could increase immunogenicity and potentiate cancer cell destruction by the immune system [46]. Although Hsps have also been implicated in cold survival, there is little direct evidence in the literature to confirm this link [47]. Discovery of the cold-inducible RNA-binding protein (CIRP) in mouse fibroblasts suggests that growth suppression at hypothermic conditions is due to an active response by the cell rather than due to passive thermal effects [48]. The gene expression changes produced by hypothermia are not fully known, but appear to differ in important ways from those produced by heat shock [49]. In this study, we did examine the expression of HSPs or CIRP, therefore, we don't know how the hypothermia affects the expression of HSPs or CIRP in vitro and vivo experiments and whether EMT and tumor immune evasion are significantly correlated with expression of HSPs or CIRP. It is possible that augmentation of anti-tumor immunity induced by HSPs or CIRP reversed the immunosuppressive microenvironment in hypothermic mice, but this remains hypothetical.

Despite substantial advances in the treatment of localized malignancies, tumor recurrence and metastasis remain the primary cause of cancer mortality [50]. In order to improve the survival of cancer patients, disseminated dormant cancer cells must be effectively eradicated to prevent tumor recurrence and metastasis. However, targeting the signals that regulate the switch from dormancy to proliferation has been hampered by the lack of recognition of the early carcinogenic microenvironment [51]. Our results suggest that hypothermia and adipocyte activation provide a favorable microenvironment for malignant progression, and targeting these early events in hypothermia and adipose activation could open new therapeutic strategies for anticancer therapy. Although the exact molecular mechanisms that hypothermia activates in adipose tissue remain unknown, previous studies have shown that symptoms of tumor patients may be alleviated markedly and that the tumor may even regress completely after a persistent high fever for several days [52]. This verifies that targeting microenvironment for tumor treatment is as important as targeting the presence of tumor. Tumor therapies should aim to stop the proliferation of tumor cells and also to stimulate a specific immune response against residual cancer cells. Progress in understanding the pivotal role of hypothermia in adipose activation makes hypothermia and adipose tissue potential targets for tumor recurrence and metastasis studies in the future.

## Materials and Methods

### Animals

Cohorts of 7- to 8-wk old female C57BL/6J mice, KKAy mice, athymic nude mice, and experimental diets were obtained from Beijing Weitong Lihua Animal Co. All mice were housed in individual ventilated cages under a 12 h light-dark cycle (lights on 7∶00 AM to 7∶00 PM). The animals were fed standard rodent chow and water, and diet-induced obese mice were fed a high-fat diet (HFD) for 5 weeks prior to experimental procedures. 12- to 13-wk old mice were used in experiments and were exposed to 24°C (normothermia) or 10°C (hypothermia) based on experimental needs. All procedures involving animals were approved by the Henan University Institutional Animal Care and Use Committee and were carried out in accordance with the Guide for the Care and Use of Laboratory Animals.

### Cell culture

Human pulmonary alveolar epithelial carcinoma cells (A549), mouse Lewis lung cancer cells (LLC), human bronchial epithelial cells (BEAS-2B), and pre-adipocytes (3T3-L1) were purchased from the Cell Bank of the Chinese Academy of Sciences in Shanghai. A549 and LLC cells were grown in RPMI1640 medium (Sigma, St. Louis, MO, USA) supplemented with 10% (v/v) fetal bovine serum (FBS, Gibco Invitrogen, Carlsbad, CA). 3T3-L1 pre-adipocytes were cultured in DMEM medium (Sigma, St Louis, MO, USA) supplemented with 10% (v/v) FBS. BEAS-2B cells were cultured in DMEM/F12 medium (Invitrogen) supplemented with 10% (v/v) FBS. 3T3-L1 cells were differentiated into adipocytes as described previously [16] and were used for experiments after full differentiation (day 14). All cells were grown in a humidified atmosphere of 5% CO2 and 95% air at 37°C or 28°C. The media from cells cultured in different temperature was collected after 24 h of incubation and centrifuged at 10,000 rpm for 10 min at 4°C, and the supernatant was stored at −80°C and used for cytokines ELISA (Quantikine, R&D Systems). Hypothermia-incubated media was defined as the media from cells cultured at 28°C, normothermia-incubated media was defined as the media from cells cultured at 37°C.

### Cell proliferation assay

BEAS-2B cells and differentiated adipocytes at 10,000 cells per well and LLC cells and A549 cells at 5,000 cells per well were seeded in a 96-well plate overnight at 37°C. Cells exposed to MNU (Sigma) for 2 h were continuously incubated for 48 h at 37°C or 28°C following addition of cytokines (R&D Systems), cytokine blocking antibody (R&D Systems), or adipocyte media. During the final 4 h of the 48 h incubation, the supernatants were disposed of, and 100 μL of MTT (0.5 mg/ml, Sigma) was added to each well. After 4 h, the MTT was disposed of, and 100 μL of DMSO was added to each well. Optical density (OD) was determined on a plate reader (BIO-IEK, Elx800) at 570 nm. Results of cell proliferation were presented as the percent of control cells.

### Soft agar assay

Cell colony formation was analyzed by soft agar assay according to the procedures of Lee K [17]. Briefly, a bottom layer of 0.5% agarose (Promega, USA) containing 2 mL of DMEM medium was initially solidified in a 6-well culture plate at 4°C. Next, 2 mL of 0.3% agarose solution containing 10,000 BEAS-2B cells treated with MNU 50 μmol/L for 2 h and cytokines, cytokine blocking antibody, or adipocyte media for 48 h, were layered on top. Each cell condition was tested in triplicate. After incubation at 37°C with 5% CO2 atmosphere for 2 weeks, the colonies that contained over 50 cells were counted under a microscope.

### Apoptosis assay

BEAS-2B cells at 5×10^5^ cells per well were seeded in a 6-well plate. After overnight incubation, the cells were pretreated with or without MNU for 2 h and cultured for 48 h. The cells were then collected, washed with PBS, and resuspended in 500 μL binding buffer, containing 10 mmol/L HEPES-NaOH (pH 7.4), 140 mmol/L NaCl, and 2.5 mmol/L CaCl2. 5 μL of Annexin V-FITC, and 5 μL of propidium iodide (PI) solution (BD Pharmingen, San Diego, CA), was added, and the cells were incubated in the dark for 15 min. Fluorescence was analyzed by flow cytometry. Early apoptosis and late apoptosis were determined as the percentage of Annexin V+/PI- cells and Annexin V+/PI+ cells, respectively. The rate of total apoptosis was the sum of early and late apoptosis. Each cell condition was tested in triplicate.

### Alkaline comet assay

The comet assay was performed under alkaline conditions according to a procedure previously described in detail [18]. Briefly, BEAS-2B cells were cultured and collected as described in the apoptosis assay. 5×10^3^ cells (20 μL) were embedded in 0.75% low-melting point agarose (80 μL) on conventional microscope slides previously coated with 1% normal melting point agarose (100 μL). After lysis in lysis buffer for 1 h at 4°C (in the dark), the slides were placed on an electrophoresis box containing an alkaline solution at pH 13, and the embedded cells were exposed to the alkali for 40 min at 4°C to allow for DNA unwinding and expression of alkali-labile damage. Electrophoresis was performed in the same buffer at 300 mA and 25 V for 15 min. After electrophoresis, the slides were neutralized and stained with 20 μg/mL PI. Images of comets were visualized with an Olympus BX60 fluorescence microscope at 200× magnification. For DNA damage analysis, Comet 5.0 (Kinetic Imaging Ltd., Liverpool, UK) was used to compute the olive tail moment (OTM) for 100 cells from each sample.

### Surgical procedures

The genetic- and diet-induced obese mice were anesthetized by an intraperitoneal injection of sodium pentobarbital (45 mg/kg). The hair was removed from the incision area, and the area was then sterilized with 70% ethanol- and betadine-soaked gauze. A short (about 1.0 cm) skin incision was made bilaterally, beginning at the lateral side of the proximal end of the hindlimb and continuing rostrally and ventrally so that the abdominal subcutaneous inguinal adipose tissue was exposed bilaterally. Roughly 2 g of the adipose tissues were separated from surrounding tissue, excised, and weighed. The muscle and skin were sutured with absorbable sutures. The sham-operated animals received a similar procedure in that the relevant adipose tissue deposit was visualized but not removed. The animals that recovered from the surgery and resumed normal behavior after 2 weeks were used for tumor allograft experiments.

### Cytotoxicity of CTL

Cytotoxic T lymphocytes (CTL, CD8+ T cells) were purified from the C57BL/6J mouse spleens using the MACS separation system (Miltenyi Biotec, Bergisch Gladbach, Germany) according to the manufacturer's instruction described in detail by Du et al [19]. Cytotoxicity of CTL was assessed using the standard ^51^Chromium-release assay. Briefly, ^51^Cr-labeled A549 cells (1×10^4^) were mixed in a 96-well microplate at the indicated E/T ratios. After 6 h of incubation, the cell-free supernatants were collected and counted on a gamma counter. The spontaneous release and total release were determined in respective wells. The total release was counted by lysing the cells with 1% Triton X-100. The percent-specific cytotoxicity was calculated using the formula:
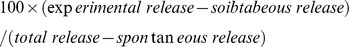



### EMT assay

BEAS-2B cells were grown to 70% confluency in a 6-well plate. The cells were then treated with control DMEM/F12, MNU, TGF-β1 or TGF-β1 blocking antibody (Invitrogen) for 48 h. The cells were collected, centrifuged, resuspended in 2 mL of cold 70% ethanol, and stored at −20°C for 24 h. After washing the cells with PBS, the cells were subsequently vortexed into a single cell suspension and incubated in PBS containing 5% BSA for 1 h to block interaction with nonspecific proteins. The cells were then incubated overnight at 4°C with primary antibodies against E-cadherin, vimentin or fibronectin (Santa Cruz Biotechnology Inc.), washed with PBS, and incubated with FITC-conjugated secondary anti-mouse IgG (Invitrogen) for 30 min at room temperature. The fluorescence signals were analyzed by flow cytometry. Geometry fluorescence was used as a measure of protein expression.

### Urethane-induced lung carcinogenesis model

To induce lung carcinogenesis, the mice housed at 24°C or 10°C were injected intraperitoneally with urethane (ethyl carbamate; Sigma, St. Louis, MO) at 800 mg/kg body weight once weekly for 8 weeks. Each group included 20 mice. The mice were sacrificed at 30 weeks to examine lung carcinogenesis, all animals entered into the study were included in the final analysis.

### Tumor allograft and xenograft experiment

The A549 and LLC cells were maintained in 10% FBS RPMI1640 medium and differentiated 3T3-L1 were maintained in 10% FBS DMEM medium until the cells reached 80% confluence. The cells were collected for tumor allograft and xenograft experiments. In the tumor allograft study, 200 μL saline containing 1×10^6^ LLC cells was injected subcutaneously into the lateral axilla of normal C57BL/6J mice, genetic-obese KKAy mice, diet-induced obese C57BL/6J mice, and fat-removed mice. Ten mice in each group were housed at 24°C or 10°C for four weeks after tumor inoculation and were then euthanized. The tumors were then removed and weighed. Animals were monitored for tumor size twice weekly.

In the tumor xenograft study, the lateral axilla of athymic nude mice was inoculated subcutaneously with saline containing 1×10^6^ A549 cells and 1×10^6^ differentiated 3T3-L1 adipocytes, cytokines or cytokine antibody (Ab), and control mice were injected with saline containing only an equivalent amount of A549 cells and adipocytes in the same location. Each group included 10 mice. Animals were monitored for tumor size at weekly intervals. After ten weeks of inoculation, mice were euthanized, and tumors were weighed.

All animals entered into the study were included in the final analysis.

The tumor volume (mm^3^) was calculated according to the following equation:
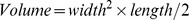



The serum was isolated for TNF-α or VEGF ELISA at the end of experiment.

## Statistical Analysis

The data were analyzed with SPSS software, version 16.0. Normally distributed continuous variables were compared between study groups with Student's t-test, and nonnormally distributed continuous variables were compared with the Mann–Whitney U test. All testing was two-sided, and a *P* value of less than 0.05 was considered to indicate statistical significance.
